# Bisphenol A-induced Alterations in Different Stages of Spermatogenesis and Systemic Toxicity in Albino Mice (*Mus musculus*)

**DOI:** 10.5696/2156-9614-11.29.210307

**Published:** 2021-02-25

**Authors:** Okunola A. Alabi, Kehinde I. Ologbonjaye, Adewale A. Sorungbe, Olutayo S. Shokunbi, Oyinkansola I. Omotunwase, Gbemisola Lawanson, Oluwafemi G. Ayodele

**Affiliations:** 1 Department of Biology, Federal University of Technology, Akure, Ondo State, Nigeria.; 2 Department of Biochemistry, School of Basic Medical Sciences, Babcock University, Ilishan-Remo, Ogun State, Nigeria.

**Keywords:** bisphenol A, sperm morphology assay, spermatogenesis, hematological parameter, biochemical parameter

## Abstract

**Background.:**

Bisphenol A (BPA) is known to alter sperm morphology, but information is limited on the most susceptible stage(s) of spermatogenesis, especially in mice.

**Objectives.:**

This study investigated the reproductive, biochemical, and hematological changes caused by exposure to BPA in male albino mice. The genotoxicity of BPA to the six stages of spermatogenesis in mice was determined.

**Methods.:**

Mice were exposed orally to BPA at 0.5, 1.0, 2.0, and 5.0 mg/kg bw doses for 5 days and assessed for sperm morphology after 35 days. Based on the result, the second group of mice was exposed to BPA at 1.0 mg/kg bw dose for 5 days, their spermatozoa were assessed for sperm morphology based on BPA exposure at the 6 maturation stages of spermatogenesis: spermatozoa, elongating spermatids, round spermatids, secondary spermatocytes, primary spermatocytes, and spermatogonia. Biochemical and hematological analyses of the blood of exposed mice were also carried out.

**Results.:**

The results showed that BPA induced concentration-dependent, significantly (p<0.05) increased sperm cell abnormalities at three of the four concentrations tested, with the exception of 0.5 mg/kg bw, in comparison with the negative control. The highest frequency of sperm aberrations was induced in spermatozoa exposed to BPA while at the primary spermatocytes. The order of induced sperm abnormality at the different stages of exposure was: primary spermatocytes > elongating spermatids > spermatozoa > spermatogonia > round spermatids > secondary spermatocytes. The results of the biochemical analysis showed significantly (p<0.05) increased serum urea, creatinine, and alanine aminotransferase (ALT) and aspartate aminotransferase (AST) activities with a concomitant decrease in total protein content at the various stages of spermatogenesis. In addition, the results for hematological parameters showed several significant (p<0.05) modulations in mice exposed to BPA.

**Conclusions.:**

These data showed that BPA is most toxic to primary spermatocytes and alterations of biochemical and hematological parameters might be the mechanisms of induced toxicity.

**Ethics Approval.:**

The Research Ethics Committee, Federal University of Technology, Akure approved the study protocols.

**Competing Interests.:**

The authors declare no competing financial interests

## Introduction

Several chemicals released into the environment are derivatives of endocrine-disrupting chemicals, which negatively affect the structure and function of the endocrine system, leading to damaging health effects. Bisphenol A (BPA) is a major endocrine-disrupting chemical and a common xenoestrogen compound which at present is one of the most utilized chemicals in the world for manufacturing many polycarbonates, polyesters, and epoxy resins.^1,2^ Bisphenol A is used in producing consumer products such as food cans, beverage cans, plastic containers, baby and water bottles, sports equipment, CDs, medical devices, and household electronics.^3,4^ It is also used in compositions of different forms of flame retardants, inner linings of food and beverage cans, paper coatings, adhesives, and dental sealants.^5^ About 67% of BPA is used in producing polycarbonate materials, 30% is applied in manufacturing epoxy resins while the remaining 3% is incorporated into other products.^6^

Globally, over six billion pounds of BPA are manufactured annually, and more than 100 tons of BPA produced are released into the atmosphere.^7^ The dietary route remains the most common route that exposes humans to BPA due to the leaching of this chemical from cans and plastic materials into various foods and drinks via hydrolysis with an increase in temperature and container age. However, temperature is the major factor that influences the leaching of BPA into foods and beverages.^8^ Other routes of exposure are through contaminated medical devices, household products, and occupational exposure such as inhalation, ingestion (during the manufacturing process), and skin contact.^9,10^

Bisphenol A has been documented to have damaging effects on the biological system. Reports have shown that blood constituents of different animals are altered after exposure to BPA. For example, studies have shown that BPA induced an increase in lymphocyte proliferation in aqua-cultured goldfish, *Carassius auratus*,^11^ and altered hematological parameters in rats.^12,13^ Estrogenic activity of BPA has been particularly linked with these injurious effects exerted by BPA.^14^ Damaging effects of BPA on the reproductive systems of males and females have been documented in rodents. Bisphenol A in adult male rats caused a lower motility and sperm count and negatively affected the overall sperm morphology.^15^ Reduction in semen quality and increased destruction of sperm DNA reported by Meeker *et al*.^16^ further buttresses germ cell toxicity by BPA. In addition, BPA has been reported to reduce the testicular and epididymal weight, epididymal and testicular sperm counts, concentration of plasma testosterone, and increase abnormal sperm morphology in rodents.^17^

**Abbreviations**
*ALT*: Alanine aminotransferase
*AST*: Aspartate aminotransferase
*MCH*: Mean corpuscular hemoglobin
*MCHC*: Mean corpuscular hemoglobin concentration
*MCV*: Mean corpuscular volume
*RBC*: Red blood cell
*WBC*: White blood cell

In rat Leydig cells, BPA has been shown to inhibit testicular steroidogenesis through the reduction of expression on genes producing steroidogenic enzymes and the luteinizing hormone secretion.^18^ Elevated abnormal morphology of sperm cells coupled with decreased motility and sperm count following two weeks of exposure to 10–40 mg/kg bw BPA concentrations have been observed.^19^ Reports have shown a decrease in sperm production^20,21^ and testosterone secretion^18^ in BPA exposed rats. In addition, epididymis size reduction, prostate duct enlargement in male fetuses, deformed acrosomes, acrosomal caps, achromosomal vesicles, and impairment of testicular function have been documented in rats and mice exposed to BPA.^22–26^

It has been reported that BPA disrupts spermatogenesis-related events like production of androgen,^18,27^ activity of Sertoli cells,^28,29^ and negative effects on the hepatic oxidative stress biomarkers in male rats as a possible mechanism of altered spermatogenesis.^30^ It is generally believed that induction of oxidative stress by toxicants is the most common cause of damage to the sperm cell,^31^ and studies have indeed shown the impact of oxidative stress on reproductive toxicity in males exposed to toxicants.^32,33^

Study of BPA toxicity on mice spermatogenesis is limited. There is a need for more studies on the toxicity of BPA in different mammals to give comprehensive toxicity information on exposure to BPA, and the possible effect of BPA on humans. Indeed, the human body-burden of BPA has been reported. In the USA, BPA was reported to be present in the serum and urine of 394 adults.^34^ This, therefore, calls for wholistic studies on its possible effect(s). Furthermore, no study has reported the spermatogenesis stage(s) that is/are most susceptible to BPA in mice. This is important to confirm if BPA acts differently on the spermatogenesis of different mammals. Knowing the specific spermatogenesis stage(s) with the highest susceptibility to BPA can help in the prevention of BPA toxicity on spermatogenesis or the development of stage-specific drugs for the treatment of infertility caused by BPA. The present study investigated the reproductive toxicity of BPA on the different stages of spermatogenesis in mice using a sperm morphology assay. The systemic effect of BPA was also studied by analyzing biochemical and hematological parameters in the exposed mice.

## Methods

Bisphenol A [2, 2-bis (4-hydroxyphenyl) propane; purity <99%] was obtained from the Qualikems company, India. Olive oil (Aceites Borges Pont, Spain) and cyclophosphamide (CAS 6055-19-2, Sigma-Aldrich Ltd, Canada) were purchased from standard suppliers. Bisphenol A was prepared using olive oil to obtain final doses of 0.5, 1.0, 2.0, and 5.0 mg/kg body weight (bw) of mice.

### Experimental animals

Male Swiss-albino mice (*Mus musculus*; 10–13 weeks old) purchased from the Department of Zoology, University of Ibadan, Oyo State, Nigeria were used for the study. The housing of the animals was carried out in well-ventilated wooden animal cages situated at the animal house of Biology Department, Federal University of Technology, Akure, Ondo State, Nigeria under 12 hours light and dark cycle at 22±0.5°C with 50% humidity. Prior to the experiment, the animals were acclimatized for 2 weeks. Mice were given clean drinking water and fed with standard mouse pelletized feed (*ad libitum*), twice daily, throughout the period that the experimented lasted. Animals that were 12–15 weeks old were used for the experiment. Animals were grouped based on their weight after the two-week acclimatization period. Animal care was observed in accordance with standard guidelines on the care and use of laboratory animals by the National Research Council (US) Committee.^35^ The Federal University of Technology, Akure, Research Ethics Committee approved the study and all protocols.

### Sperm morphology assay

The sperm morphology assay was carried out using previously reported modified protocol.^36,37^ Two sets of investigations were conducted. In the first experiment to investigate the reproductive toxicity of BPA using sperm morphology assay, 5 mice/group and 6 groups of mice were exposed daily to oral administration of 0.3 mL of BPA at 0.5, 1.0, 2.0, and 5.0 mg/kg bw doses for five consecutive days. Olive oil (used to dissolve BPA) was used as the negative control while 20 mg/kg bw of cyclophosphamide served as the positive control according to the Organisation for Economic Cooperation and Development (OECD) guidelines for testing of chemicals, Draft TG 483.^38^ The concentrations of BPA were chosen after a preliminary acute toxicity study. After the 5 days of treatment, the animals were allowed to complete 35 days from the first day of treatment with BPA (mice spermatogenesis is known to require about 35 days to complete).^39^ Mice were sacrificed by cervical dislocation and their caudal epididymis was excised surgically. Normal physiological saline was used in mincing the epididymis to prepare sperm suspensions. The grease-free slides were used to prepare smears after staining with eosin Y (1%) for 45 minutes. Six slides were prepared per animal (3 slides per epididymis), and five slides at random were observed at ×100 under oil immersion objective, after being air-dried and duly labeled. Analysis of one thousand sperm cells per mouse for possible abnormal morphology was carried out using the criteria of Wyrobek and Bruce.^40^ All the slides were scored blind by the same person.

In the second experiment to investigate the stage(s) of spermatogenesis most susceptible to BPA damage, mice of two groups (18 mice/group) were daily given 1.0 mg/kg bw of BPA (doses were chosen based on the positive response in the first experiment) by oral administration and olive oil (negative control) for five consecutive days, except the group sacrificed 24-hrs after the first day of injection. Three mice per group were sacrificed at 1, 5, 15, 18, 25, and 35 day(s) corresponding to BPA exposure at the maturation stages of spermatozoa, elongating spermatids, round spermatids, secondary spermatocytes, primary spermatocytes, and spermatogonia, respectively.^41^ The sperm morphology study was carried out on the spermatozoa of all the mice, which were exposed to BPA while at the different stages of maturation. Slide preparation and scoring were as described in the first experiment.

### Sperm count

The same animals used in the two experiments of sperm morphology assay were used for sperm count. Caudal epididymis removed surgically from the mice were minced in normal saline. Neubauer's hematocytometer red blood cell (RBC) counting chamber was used for sperm count from the suspension at 400x. The result was pooled for all the mice/group and then reported as mean sperm count/mL of suspension.^42^

### Biochemical analysis

Mice of the second experiment were used for this analysis. The cardiac puncture was used to collect blood into an Eppendorf tube (1.5 mL), centrifuged for 5 mins at 3000 rpm and serum aspartate aminotransferase (AST) and alanine aminotransferase (ALT) activities, creatinine, urea, and total protein were quantified following manufacturer's instructions using commercially available analyzing kits (Randox Laboratories Diagnostics Ltd, UK).

### Hematological analysis

Mice's blood from the first experiment collected through the cardiac puncture into ethylenediaminetetracetic acid (EDTA) specimen bottles were used for the analysis of various hematological parameters. An automatic blood analyzer was used to analyze RBC and white blood cells (WBC) count, mean corpuscular hemoglobin concentration (MCHC), mean corpuscular volume (MCV), hemoglobin concentration (Hb), mean corpuscular hemoglobin (MCH), platelet (PLT), hematocrit (HCT), granules (GR), and differential white blood cell count such as eosinophils, monocytes, and lymphocytes.

### Statistical analysis

Statistical Package for the Social Sciences (SPSS®) version 22.0 was used for data analyses. The results were presented as mean ± standard error and percentage frequency. Duncan's New Multiple Range Test (DMRT) and one-way analysis of variance (ANOVA) were used to test significance at different concentrations. Significant differences between the treatment groups and negative control were determined at *p* < 0.05.

## Results

Table 1 shows the result of exposure to different doses of BPA on the sperm morphology of mice after 5 consecutive days of treatment. The results showed significantly (*p* < 0.05) increased sperm cell abnormalities at all the doses tested with the exception of 0.5 mg/kg bw. The induced sperm abnormalities were dose-dependent, with the highest dose (5.0 mg/kg bw) inducing the highest sperm abnormality (302.13), compared with the negative control (65.29).

**Table 1:** Summary of Abnormal Sperm Morphologies Induced by Different Doses of Bisphenol A in Mice over Five Days

**Doses (mg/kg bw)**	**Mean±SE of abnormalities**	**% Frequency of abnormalities**
Olive oil	65.29±0.02	5.32
0.5	81.29±0.08	14.98
1.0	200.00±0.21*	39.43*
2.0	242.00±0.41*	52.32*
5.0	302.13±0.32*	61.47*
Cyclophosphamide	314.71±6.36*	65.06*

^*^ - Significant at *p*<0.05 in comparison with negative control; bw = body weight; SE = standard error of mean; olive oil = negative control; Cyclophosphamide (20 mg/kg bw) = positive control; n = 5.

In Table 2, the mean of sperm morphology abnormalities induced in spermatozoa treated with BPA at a dose of 1.0 mg/kg bw at the different stages of spermatogenesis in mice revealed that BPA is toxic to all the stages of spermatogenesis. Bisphenol A induced significantly (*p* < 0.05) increased sperm cell abnormalities after exposure to all the stages of spermatogenesis which at least doubled their corresponding negative control. However, the highest frequency of sperm cell abnormalities was induced when spermatozoa were exposed at primary spermatocytes while the least was at secondary spermatocytes. The order of induced sperm abnormality in spermatozoa at the different stages of development was primary spermatocytes > elongating spermatids > spermatozoa > spermatogonia > round spermatids > secondary spermatocytes.

**Table 2:** Summary of Abnormal Sperm Morphologies Induced in Mice Spermatozoa Exposed to Bisphenol A at Different Stages of Spermatogenesis

**Stages of spermatogenesis**	**Mean±SE of abnormalities (1 mg/kg BPA)**	**Mean±SE of abnormalities (Olive oil)**
Cyclophosphamide	327.00±0.1 l^a*^	
Spermatogonia (35 days)	209.33 ±0.72^b*^	52.21±0.20
Primary spermatocytes (25 days)	301.33±0.79^a*^	58.11±0.10
Secondary spermatocytes (18 days)	174.00±0.44^c*^	55.61±0.05
Round spermatids (15 days)	179.33±0.78^c*^	60.01±0.62
Elongating spermatids (5 days)	256.33±0.41^d*^	63.82±0.11
Spermatozoa (1 day)	228.33±0.25^e*^	65.00±0.53

^*^ - Significant at *p*<0.05 in comparison with negative control; BPA - Bisphenol A; SE = standard error of mean; values with similar alphabets are not significantly different; Olive oil = negative control; Cyclophosphamide = positive control; n = 3.

**Figure 1:**
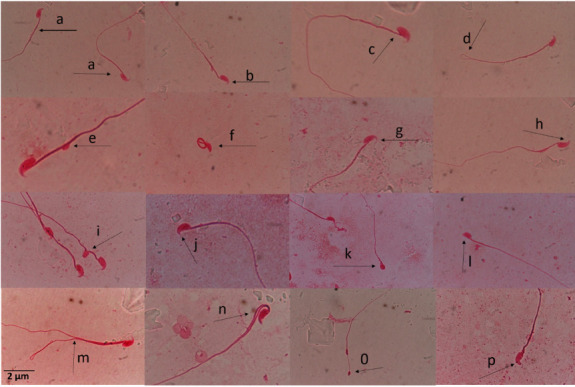
Sperm morphology abnormalities induced in BPA-treated mice (a) sperm cell with normal morphology, (b) knobbed hook, (c) tail at wrong angle, (d) looped tail, (e) distal droplet, (f) folded sperm, (g) knobbed hook, (h) hook at wrong angle, (i) fused neck, (j) short hook, (k) amorphous head, (l) no hook, (m) double tail, (n) split tail, (o) pin head with extension, and (p) kidney shape sperm. (1% Eosin Y stain; ×100).

Sperm aberrations such as sperm cell with double tail, amorphous head, pin head with extension, kidney-shaped head, short hook, knobbed hook, no hook, wrong tail attachment, distal droplet, looped tail, fused neck, double head, split tail and folded sperms were observed *(Figure 1).* Sperm cell with amorphous head had the highest frequency of aberration in exposed mice while sperm cells with split tails had the least frequency. Food consumption and body weight of BPA-exposed mice were not significantly affected throughout the study period.

### Sperm count

The results of the mean sperm count recorded from mice treated with different BPA doses and at different stages of spermatogenesis are presented in Table 3. Means of 4.63 × 10^8^ and 48.59 × 10^6^ were observed for negative and positive controls, respectively. However, BPA at 0.5, 1.0, 2.0, and 5.0 mg/kg bw doses induced a statistically significant (*p* < 0.05), dose-dependent reduction of 3.79 × 10^8^, 86.04 × 10^7^, 34.50 × 10^7^, and 54.33 × 10^6^ sperm cells/mL, respectively, in the exposed mice. Similar to the result of the abnormal sperm morphology, 1.0 mg/kg bw of BPA induced a significant (*p* < 0.05) reduction in the mean sperm cell count at the different stages of spermatogenesis in mice in the order: primary spermatocytes < elongating spermatids < spermatozoa < round spermatids < secondary spermatocytes < spermatogonia. The mean/mL of sperm cells at the different stages of spermatogenesis is between 81.05 × 10^7^ and 85.62 × 10^7^.

**Table 3:** Mean of Sperm Count in Mice Treated with Different Doses of Bisphenol A at Different Stages of Spermatogenesis

**Doses (mg/kg bw)**	**Mean sperm count (/mL)**

Olive oil	4.63 × 10^8^
0.5	3.79 × 10^8^
1.0	86.04 × 10^7*^
2.0	34.50 × 10^7*^
5.0	54.33 × 10^6*^
Cyclophosphamide	48.59 × 10^6*^

**Stages of spermatogenesis**	

Spermatogonia (35 days)	85.62 × 10^7*^
Primary spermatocytes (25 days)	81.05 × 10^7*^
Secondary spermatocytes (18 days)	85.52 × 10^7*^
Round spermatids (15 days)	84.47 × 10^7*^
Elongating spermatids (5 days)	82.07 × 10^7*^
Spermatozoa (1 day)	84.00 × 10^7*^

^*^ - Significant at *p<*0.05 in comparison with negative control; BPA - Bisphenol A; bw - body weight; Cyclophosphamide = positive control (20 mg/kg bw); Olive oil = negative control.

### Biochemical analysis

Table 4 shows the effects of BPA on serum urea, creatinine, total protein, and activities of ALT and AST in treated mice at each stage of spermatogenesis. The observed data showed significantly (*p* < 0.05) increased serum AST, creatinine and urea at all the stages of spermatogenesis in mice, while serum ALT increased significantly (*p* < 0.05) at primary spermatocytes, round and elongating spermatids, and spermatozoa in comparison to the negative control. Furthermore, BPA induced a significant (*p* < 0.05) decrease in total protein at spermatogonia, primary and secondary spermatocytes and spermatozoa stages in comparison to the negative control.

**Table 4:** Biochemical Parameters in Serum of Mice Exposed to Bisphenol A at Different Stages of Spermatogenesis

Parameters	Olive oil	35 days	25 days	18 days	15 days	5 days	1 day

ALT (U/L)	65.70±1.90	67.60±1.90	87.10±3.10*	67.55±4.75	95.25±0.85*	78.90±4.50*	70.20±2.30*
AST(UL)	307.10±4.50	349.45±5.25*	387.10±8.70*	385.55±1.85*	394.70±5.80*	363.70±0.50*	367.35±1.65*
Creatinine (mg/dL)	0.20±0.01	0.26±0.01*	0.28±0.02*	0.28 ±0.01*	0.27±0.02*	0.26±0.04*	0.23±0.10*
Urea (mg/dL)	24.00±1.00	28.20±0.80*	27.60±2.60*	32.40±0.80^b*^	33.10±1.10*	36.80±1.70*	30.30±0.10*
Total protein (mg/dL)	7.17±35	4.69 ±0.17*	5.08±0.73*	5.51±0.30*	5.59±0.99	6.69 ±0.36	5.05±0.30*

^*^ *-* Significant at *p*<0.05 in comparison with negative control; olive oil = negative control; AST = aspartate aminotransferase; ALT = alanine aminotransferase; 35 days - spermatogonia; 25 days - primary spermatocytes; 18 days - secondary spermatocytes; 15 days - round spermatids; 5 days - elongating spermatids; 1 day - spermatozoa; values represent mean±standard error of mean.

### Hematological analysis

The results of the effect of different doses of BPA on hematological parameters in mice is presented in Tables 5a and 5b Some of the doses of BPA induced significantly (*p*<0.05) increased WBC, RBC, granulocytes, lymphocytes, platelets, MCH, MCV, and MCHC with a concomitant significant reduction (*p* < 0.05) in monocytes, hemoglobin, and HCT. However, eosinophils were not significant (*p* > 0.05). All the stages of spermatogenesis had a significant increase in WBC, granulocytes, lymphocytes, RBC, MCV, platelets, and MCHC, but a significant decrease in monocytes, hemoglobin, and HCT. Both eosinophils and MCH were not significant (*p* > 0.05) in comparison to the negative control.

**Table 5a:** Hematological and Immunological Parameters of Bipshenol A-exposed Mice at Different Stages of Spermatogenesis

	WBC	GRAN (%)	LYM (%)	Mono (%)	EOS (%)	PLT (×10^9^/L)
1^st^						
Olive	4.80±0.33	27.30±0.88	50.70±0.84	20.70±0.18	1.30±0.33	3.50±0.11
0.5	6.20±0.20*	29.50±0.42	52.90±0.01	22.90±0.08	1.20±0.07	3.70±0.10
1.0	8.20±0.23*	55.00±0.36*	28.00±1.00*	16.00±0.65*	1.00±0.00	5.60±1.21*
2.0	9.70±1.63*	31.70±2.85	54.70±0.06	12.30±1.86*	1.30±0.33	3.80±0.59
5.0	9.90±0.06*	44.50±0.50*	39.50±0.50*	14.50±0.50 *	1.50±0.50	4.20±1.00*
CP	8.40±0.94*	33.70±0.33	49.00±1.03*	16.30±0.88*	1.00±0.00	3.20±0.60
2^nd^						
Olive	4.60±0.22	26.80±0.03	51.20±0.38	19.90±1.01	1.20±0.11	3.40±0.92
35 days	6.20±0.92*	29.00±1.06*	58.00±4.00*	11.30±2.33*	1.70±0.33	5.90±2.21*
25 days	9.50±3.43*	25.30±9.82*	65.00±2.58*	8.30±3.18*	1.30±0.33	4.60±0.13*
18 days	8.50±0.99*	10.00±1.00*	80.50±0.50*	8.00±1.00 *	1.50±0.50	5.20±5.00*
15 days	6.70±1.91*	8.70±2.67*	72.00±1.53*	17.70±4.33*	1.30±0.33	5.30±1.08*
5 days	6.20±3.32*	14.50±6.50*	76.50±1.50*	7.50±6.50*	1.50±0.50	4.60±2.19*
1 day	8.30±0.68*	31.70±1.38*	61.70±1.71*	5.70±1.33*	1.30±0.33	5.80±9.83*

Abbreviations: CP, cyclophosphamide; EOS, eosinophils; GRAN, granulocytes; LYM, lymphocytes; Mono, monocytes; Olive, olive oil; PLT, platelets; WBC, white blood cell.

Concentrations of bisphenol A are in mg/kg bw; Negative control - Olive oil; Positive control - Cyclophosphamide (20 mg/kg bw);

^*^ - Significant at *p<*0.05 in comparison with negative control. 35 days - spermatogonia; 25 days - primary spermatocytes; 18 days - secondary spermatocytes; 15 days - round spermatids; 5 days - elongating spermatids; 1 day - spermatozoa; values represent mean±standard error of mean.

**Table 5b:** Hematological and Immunological Parameters of Bipshenol A-exposed Mice at Different Stages of Spermatogenesis

	RBC (×10^12^/L)	MCH (pg)	HGB (g/L)	HCT (%)	MCV (fl)	MCHC (g/dl)
1^st^						
Olive	6.80±0.67	14.00±0.46	13.20±0.43	39.30±0.33	43.70±0.03	32.70±1.01
0.5	7.00±0.28	14.50±0.50	13.00±0.32	38.70±0.01	44.90±0.22*	32.90±0.22
1.0	8.10±0.43*	16.20±0.23*	13.10±0.85	38.30±2.03	48.70±0.67*	33.20±0.25*
2.0	8.50±0.23*	14.60±0.31	12.70±0.27*	37.30±0.33*	43.30±0.88	34.40±0.52*
5.0	7.20±0.20	15.50±1.15*	11.00±0.60*	32.50±2.50*	46.50±2.50*	33.10±0.65*
CP	8.10±0.55*	14.80±1.76	11.90±0.98*	39.20±1.24	48.30±1.33*	30.70±3.17*
2^nd^						
Olive	6.20±1.01	14.30±0.05	13.50±0.80	39.90±0.60	43.20±0.28	32.10±0.61
35 days	7.10±0.49*	14.70±0.88	10.40±1.01*	37.70±4.06*	53.30±5.70*	27.60±1.71*
25 days	7.80±0.88*	14.90±0.09	11.60±1.30*	36.00±4.36*	46.30±1.45*	32.20±0.87
18 days	8.80±0.16*	14.50±0.60	12.80±0.75*	36.50±1.50*	41.50±1.50*	34.70±0.20*
15 days	6.50±1.09	14.50±0.62	9.50±1.68*	29.30±5.24*	45.70±1.20*	31.70±0.49*
5 days	6.60±2.16	14.60±0.10	9.60±3.05*	29.50±8.50*	45.00±1.00*	32.30±0.70
1 day	7.50±0.42*	13.70±0.50	10.30±0.95*	32.00±2.00*	41.00±1.16*	33.50±0.30*

Abbreviations: CP, cyclophosphamide; HCT, hematocrit; HGB, hemoglobin; MCH, mean corpuscular hemoglobin; MCHC, mean corpuscular hemoglobin concentration; MCV, mean corpuscular volume; Olive, olive oil; RBC, red blood cell.

Concentrations of bisphenol A are in mg/kg bw; Negative control - Olive oil; Positive control - Cyclophosphamide (20 mg/kg bw)

^*^ - Significant at *p<*0.05 in comparison with negative control. 35 days - spermatogonia; 25 days - primary spermatocytes; 18 days - secondary spermatocytes; 15 days - round spermatids; 5 days - elongating spermatids; 1 day - spermatozoa; values represent mean±standard error of mean.

## Discussion

One of the major reproductive disorders of public health concern is male infertility. This form of reproductive disorder has been associated with estrogenic compounds like BPA.^43,44^ In addition, BPA is known to interfere with androgenic production which is a process related to spermatogenesis and alteration of hematological parameters.^12^ This present research provided information on BPA toxicity in Swiss albino mice using reproductive, biochemical, and hematological approaches. The result of the sperm morphology assay in this study confirmed previous reports that BPA is a germ cell mutagen by the induction of a significant increase in mice sperm abnormalities. A significant increase in abnormal sperm morphology frequency such as that reported in the present study is a major cause of male infertility.^45^

Our data further confirmed that BPA is mutagenic to the six stages of spermatogenesis in mice, inducing significant damage at all the stages of spermatogenesis, which eventually produces different sperm abnormalities of the final spermatozoa. However, the results showed that primary spermatocytes were the most sensitive stage of mouse spermatogenesis to BPA, recording the highest increase in abnormal sperm cells in mice after completion of spermatogenesis. This suggests that BPA exerted the most damaging effects on differentiated spermatogonia or on the interface between spermatocytes and differentiated spermatogonia in mice. This result agrees with the belief that spermatocytes are largely susceptible to chemotherapeutic agents, heat shock, growth factor deprivation, and ionizing radiation.^46–48^

Bisphenol A can induce sperm abnormalities by crossing the blood-testis barrier and interfere with the growth and development of sperm in exposed mice.^49^ Bisphenol A damage to spermatocytes may be caused by the alteration of nuclear structure and broken nuclear membrane.^50^ The effect of BPA in elongating spermatids in this study may be a result of abnormalities in acrosomal vesicles and abnormal nuclear morphology such as condensed granules of sperm chromatin with the damaged nuclear envelope.^50,51^ Allen *et al.*^52^ reported that spermatids are sensitive to clastogenic agents such as BPA due to chromatin contraction during the post-meiotic period of the spermatogenic cycle. Furthermore, spermatids are specifically sensitive to chromatic damaging agents probably as a result of the loss of DNA repair enzymes at the later spermatogenesis stages.^53^ The effects of BPA on the spermatocytes and spermatids in this study partially agrees with the report of Tiwari and Vanage,^54^ where spermatids and spermatocytes of adult male rats exposed to BPA were the most sensitive stages of spermatogenesis; however, in the present study, primary spermatocytes and not spermatids was the most sensitive stage. This observed difference might be due to the type of rodents used in the two studies, rats in the study by Tiwari and Vanage^54^ and mice in the present report.

The results reported in the present study further revealed that BPA induced several types of abnormal sperm morphology such as sperm head and midpiece defects (double head, amorphous head, pinhead, kidney-shaped head, short hook, knobbed hook, no hook, and swollen mid-piece), and sperm tail defects (double tail, wrong tail attachment, distal droplet, split tail, bent neck, and folded sperm). Sperm head and midpiece defects have been classified as primary defects of spermatogenesis. These primary defects are more likely to be associated with decreased fertility.^55^ Head defect abnormalities such as pin, kidney-shaped, double head, and amorphous heads observed in this study might not affect sperm cell's motility but is capable of utterly reducing their capacity to fertilize both *in vivo* and *in* vitro.^56^ Sperm head defect with pin-like structure is usually formed without integration of DNA into their own heads. Their small head is too narrow to execute effective fertilization. Sperm with double head as presented in this study may cause a reduction in sperm motility and makes it difficult for sperm to penetrate an egg during fertilization.^57^ Sperm cells with abnormal hook (short hook, long hook, and hook at the wrong angle) are mostly believed to have occurred as a result of the disorganized arrangement of the membrane of the acrosome leading to change in the shape of the nucleus. Sperm head defects also served as biomarkers of other abnormalities in sperm cells with a significant ability of fertility disruption.^58^ The sperm head defects reported in this study might be due to the effects of BPA on genes which function in expressing acrosome characteristics.^59^ Studies by Agunbiade *et al.*^60^ and Odeigah *et al.*^61^ showed that abnormalities of sperm heads could occur as a result of genetic mechanisms or testicular DNA alterations from cytotoxic agents, which ultimately alters events leading to spermatozoa differentiation.

Sperm tail defects (short tail, wrong tail attachment, and folded tail) might be an indication of aging of sperm cells, while bent mid-piece defects possibly occurred from abnormal centrioles. Sperm tail defects mostly occur during the storage of sperm, maturation, and epididymal transit during which motility of spermatozoa develops.^62^ Folded sperm cell, which was the most commonly induced sperm abnormality in this study correlates with the report of Bakare *et al.*^63^ who reported folded sperm as the highest frequency of occurrence in mice following exposure to nanoparticles. Folded sperm cannot swim to fertilize an egg as the tail is coiled around the mid-piece.

In addition, this study showed a significant mean sperm count reduction in BPA-treated mice in comparison to controls. It is an indication that BPA does not only alter the quality of sperm cells, but also significantly reduces sperm cell quantity in mice during spermatogenesis.^64^ Reduction of sperm quantity in these mice may be due to the damaging ability of BPA on Sertoli cells whose population is a determining factor of the sperm cells volume that will be produced. A decrease in the number of viable sperm cells in an organism may be an additional factor in male infertility.^42^

The potential mechanism for the altered spermatogenesis induced by BPA was investigated through liver and kidney biomarkers. Our results showed significantly increased activities of ALT and AST in BPA-treated mice during the different stages of spermatogenesis. Alanine transaminase and AST are considered sensitive indicators of stress, and markers of hepatocyte injury.^65^ The significant increase in the activities of ALT and AST by BPA in this report agrees with reports by Mourad and Khadrawy^66^ and Mahdavinia *et al.*,^67^ where BPA was shown to induce significantly increased ALT and AST in rats. In addition, similar to our report of significant elevation of serum creatinine and urea is the report of Rahimi *et al.*,^68^ where BPA was reported to cause elevated levels of serum creatinine and urea after intraperitoneal administration of BPA for 15 days. An increase in these parameters may lead to chronic renal failure which has been reported to be linked with testicular damage and altered spermatogenesis which generally leads to infertility.^69^ High levels of urea and creatinine also leads to advanced uremia and it has been reported to cause spermatogenic damage with evidence of a reduced number of spermatocytes,^70^ and high frequency of infertility in uremic rats.^71^ The data from this research showed a decrease in total protein content in BPA-treated mice in comparison to the negative control, probably as a result of alterations caused by BPA to deactivate the protein disulfide isomerase, responsible for the folding and release of cellular proteins.^72^ The decreased protein content could also be related to the decreased ability of hepatocytes to produce necessary proteins for transport and other metabolic roles as caused by BPA treatment. A similar observation of a significant decrease in protein content of BPA-treated rats has also been documented.^73^

When BPA enters into the biological system, it is either directly or subsequently absorbed into the blood stream depending on the route of entry, therefore, the assessment of hematological and immunological parameters to provide the state of blood constituent after exposure to BPA becomes important in toxicological studies. Results from this study showed significantly increased WBC, RBC, granulocytes, lymphocytes, platelets, MCH, MCV and MCHC with a concomitant significant reduction in monocytes, hemoglobin, and HCT. Hematological parameters including RBC, HGB, WBC, and hematological indices like MCHC, MCHC, and MCV are generally regarded as strong bio-indicators of cellular toxicity.^74^ An increase in RBC may indicate the stimulation of erythropoiesis. Severe stimulation of erythropoiesis has been reported to induce an increase in the frequency of damage to the DNA in the blood cells.^64^ Platelets are useful in wound healing, the body's immune defense and very important in the different stages of blood coagulation,^75^ therefore, an increase in platelets in this study may indicate deleterious effects of BPA on the immune system. An increased level of blood WBC indicates an increase in an organism's defensive ability against potential infections, and it is responsible for both specific and non-specific immunity. The increase in the concentration of WBC as presented in this study may also indicate stimulation of the immune system in response to damage caused by BPA. Similar to our report, hematological and immunological alterations by BPA in rats^12,13,76^ have also been reported. Previous reports have suggested that BPA might have a complex immune-modulating effect by stimulating or suppressing the immune system. For example, studies^77–79^ have shown that BPA caused alteration in antibody production and modulates the production of T-helper cells in exposed rats.

## Conclusions

In conclusion, this study has shown that BPA is a potential germ cell mutagen, has the ability to impair the process of spermatogenesis most likely through liver and kidney damage, and also alters various hematological indices in mice. In the present study, primary spermatocytes, elongating, and round spermatid stages of spermatogenesis were the most sensitive to BPA. At these stages, alterations in liver and kidney biomarkers induced in BPA-treated mice indicated that liver and kidney damage might be mechanisms of induced toxicity. The development of a stage-specific drug that will be effective in treating the affected stage(s) of spermatogenesis in humans exposed to BPA is recommended. In addition, an awareness campaign on the effect of BPA on male fertility and hematotoxicity should be promoted among the general public. Finally, further study is needed to understand the potential effects of human exposure to BPA using various exposure windows of interest including dosage, administration route, and period of exposure.
